# Comparison of Diagnostic Criteria for Common Variable Immunodeficiency Disorder

**DOI:** 10.3389/fimmu.2014.00415

**Published:** 2014-09-15

**Authors:** Rohan Ameratunga, Maia Brewerton, Charlotte Slade, Anthony Jordan, David Gillis, Richard Steele, Wikke Koopmans, See-Tarn Woon

**Affiliations:** ^1^Department of Virology and Immunology, Auckland Hospital, Auckland, New Zealand; ^2^Department of Clinical Immunology, Auckland Hospital, Auckland, New Zealand; ^3^Department of Clinical Immunology, Royal Melbourne Hospital, Melbourne, VIC, Australia; ^4^Department of Clinical Immunology, Royal Brisbane Hospital, Brisbane, QLD, Australia

**Keywords:** common variable immunodeficiency, diagnostic criteria, HGUS

## Abstract

Common variable immunodeficiency disorders (CVIDs) are the most frequent symptomatic primary immune deficiency condition in adults. The genetic basis for the condition is not known and no single clinical feature or laboratory test can establish the diagnosis; it has been a diagnosis of exclusion. In areas of uncertainty, diagnostic criteria can provide valuable clinical information. Here, we compare the revised European society of immune deficiencies (ESID) registry (2014) criteria with the diagnostic criteria of Ameratunga et al. (2013) and the original ESID/pan American group for immune deficiency (ESID/PAGID 1999) criteria. The ESID/PAGID (1999) criteria either require absent isohemagglutinins or impaired vaccine responses to establish the diagnosis in patients with primary hypogammaglobulinemia. Although commonly encountered, infective and autoimmune sequelae of CVID were not part of the original ESID/PAGID (1999) criteria. Also excluded were a series of characteristic laboratory and histological abnormalities, which are useful when making the diagnosis. The diagnostic criteria of Ameratunga et al. (2013) for CVID are based on these markers. The revised ESID registry (2014) criteria for CVID require the presence of symptoms as well as laboratory abnormalities to establish the diagnosis. Once validated, criteria for CVID will improve diagnostic precision and will result in more equitable and judicious use of intravenous or subcutaneous immunoglobulin therapy.

## Introduction

Common variable immunodeficiency disorders (CVIDs) are the most frequent symptomatic primary immune deficiency disorder in adults. While most patients suffer recurrent infections (1–5), there is also an increased risk of autoimmune disorders and malignancy because of immune dysregulation. CVID is likely to represent a heterogeneous group of polygenic disorders (6).

Although some patients have symptoms dating back to early childhood (7), the hallmark of CVID is primary hypogammaglobulinemia, which is a consequence of late onset antibody failure (LOAF). The majority of adult patients with CVID have IgG levels below 5 g/l (5). Most patients also have reduced or undetectable IgA and/or IgM levels (1, 8). Other laboratory features may include reduced switched memory B cells and/or increased numbers of CD21 low B cells in the periphery (9). A subgroup of patients with severe T-cell defects, originally included within the spectrum of CVID, are now defined separately as late onset combined immune deficiency (LOCID) (10).

In addition to the laboratory features outlined above, many patients have characteristic histological lesions including a sarcoidosis-like granulomatous disorder, nodular lymphoid hyperplasia of the gut, nodular regenerative hyperplasia of the liver, or lymphoid interstitial pneumonitis of the lungs (11–16). Absence of plasma cells in gastrointestinal biopsies is another characteristic finding in the majority of CVID patients (17–19).

## Diagnosis of CVID

It can be very difficult to determine which patients are suffering from CVID and require intravenous immunoglobulin (IVIG) or subcutaneous immunoglobulin (SCIG) replacement. IVIG/SCIG treatment can substantially improve both quality of life (20) and longevity in patients with CVID. This underscores the importance of accurate diagnostic criteria for CVID (21).

Here, we compare the original (1999) European society of immune deficiencies (ESID)/pan American group for immune deficiency (PAGID) criteria, the Ameratunga et al. (2013) criteria and the revised ESID registry (2014) criteria (Tables 1–3). We have previously described in detail the evidence base for the Ameratunga et al. (2013) criteria (21). It is necessary to provide an abbreviated version of these criteria here to allow comparison with the revised ESID registry (2014) criteria. We have devoted similar space to each of these criteria.

**Table 1:** **The original ESID/PAGID (1999) criteria for probable and possible CVID**.

Probable
**Probable CVID**
Male or female patient who has a marked decrease of IgG (at least 2 SD below the mean for age) and a marked decrease in at least one of the isotypes IgM or IgA, and fulfils all of the following criteria:
∙ Onset of immunodeficiency at >2 years of age
∙ Absent isohemagglutinins and/or poor response to vaccines
∙ Defined causes of hypogammaglobulinemia have been excluded
**Possible CVID**
Male or female patient who has a marked decrease (at least 2 SD below the mean for age) in at least one of the major isotypes (IgM, IgG, and IgA) and fulfils all of the following criteria:
1. Onset of immunodeficiency at >2 years of age
2. Absent isohemagglutinins and/or poor response to vaccines
3. Defined causes of hypogammaglobulinemia have been excluded

**Table 2:** **New diagnostic criteria (Ameratunga et al., 2013) for CVID**.

**A**	Must meet all major criteria
	∙ Hypogammaglobulinemia IgG < 5 g/l (5)
	∙ No other cause identified for immune defect (22)
	∙ Age > 4 years (23)
**B**	Sequelae directly attributable to immune system failure (ISF) (one or more)
	∙ Recurrent, severe, or unusual infections
	∙ Poor response to antibiotics
	∙ Breakthrough infections in spite of prophylactic antibiotics
	∙ Infections in spite of appropriate vaccination, e.g., HPV disease
	∙ Bronchiectasis and/or chronic sinus disease
	∙ Inflammatory disorders or autoimmunity (24)
**C**	Supportive laboratory evidence (three or more criteria)
	∙ Concomitant reduction or deficiency of IgA (<0.8 g/l) and/or IgM (0.4 g/l) (1, 4)
	∙ Presence of B cells but reduced memory B cell subsets and/or increased CD21 low subsets by flow cytometry (9)
	∙ IgG3 deficiency (<0.2 g/l) (25, 26)
	∙ Impaired vaccine responses compared to age-matched controls (27)
	∙ Transient vaccine responses compared with age-matched controls (28, 29)
	∙ Absent isohemagglutinins (if not blood group AB) (30)
	∙ Serological evidence of significant autoimmunity, e.g., Coombs test
	∙ Sequence variations of genes predisposing to CVID, e.g., *TACI, BAFFR, MSH5*, etc. (31, 32)
**D**	Presence of relatively specific histological markers of CVID (not required for diagnosis but presence increases diagnostic certainty, in the context of category A and B criteria)
	∙ Lymphoid interstitial pneumonitis (11)
	∙ Granulomatous disorder (12, 13)
	∙ Nodular regenerative hyperplasia of the liver (14, 15)
	∙ Nodular lymphoid hyperplasia of the gut (16)
	∙ Absence of plasma cells on gut biopsy (17, 18)

**Figure 1:**
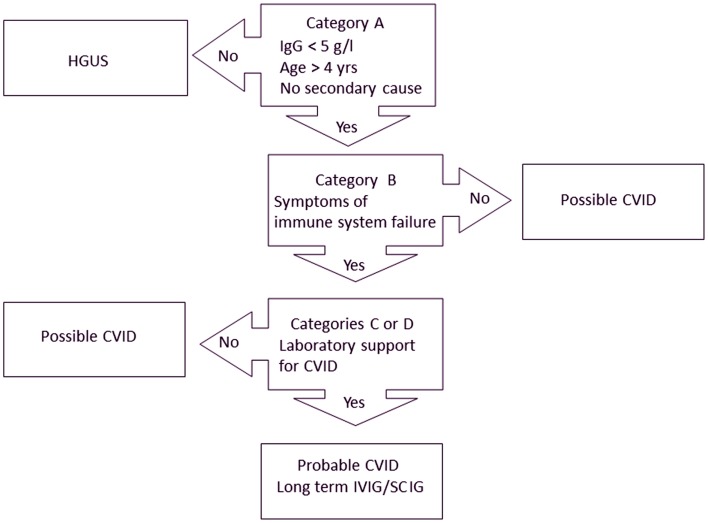
**Treatment algorithm for CVID (21)**. Patients must meet all major criteria in category A for consideration of CVID. Category B confirms the presence of symptoms indicating immune system failure (ISF). To have probable CVID, patients must also have supportive laboratory evidence of immune system dysfunction (category C) or characteristic histological lesions of CVID (category D). Patients with mild hypogammaglobulinemia (IgG > 5 g/l) are termed hypogammaglobulinemia of uncertain significance (HGUS). Patients meeting category A criteria but not other criteria are deemed to have possible CVID. Most patients with probable CVID are likely to require IVIG/SCIG. Some patients with possible CVID who have profound hypogammaglobulinemia will require IVIG/SCIG but most patients with HGUS are unlikely to need IVIG/SCIG replacement.

*Meeting criteria in categories ABC or ABD indicates probable CVID. Patients meeting criteria ABC and ABD should be treated with IVIG/SCIG (Figure 1). Patients meeting criteria A alone, AB or AC or AD but not B, are termed possible CVID. Some of these patients may need to be treated with IVIG/SCIG. Patients with levels of IgG > 5 g/l, not meeting any other criteria are termed hypogammaglobulinemia of uncertain significance (HGUS) (21). These diagnostic criteria must be applied sequentially as none are specific individually*.

**Table 3:** **Revised ESID (2014) diagnostic criteria for CVID**.

At least one of the following:
∙ Increased susceptibility to infection
∙ Autoimmune manifestations
∙ Granulomatous disease
∙ Unexplained polyclonal lymphoproliferation
∙ Affected family member with antibody deficiency
**AND** marked decrease of IgG and marked decrease of IgA with or without low IgM levels (measured at least twice; <2 SD of the normal levels for their age);
**AND** at least one of the following:
∙ Poor antibody response to vaccines (and/or absent isohemagglutinins); i.e., absence of protective levels despite vaccination where defined
∙ Low switched memory B cells (<70% of age-related normal value)
**AND** secondary causes of hypogammaglobulinemia have been excluded (see separate list)
**AND** diagnosis is established after the fourth year of life (but symptoms may be present before)
**AND** no evidence of profound T-cell deficiency, defined as two out of the following (y = year of life):
∙ CD4 numbers/microliter: 2–6 y < 300, 6–12 y < 250, >12 y < 200
∙ % Naive CD4: 2–6 y < 25%, 6–16 y < 20%, >16 y < 10%
∙ T-cell proliferation absent

*http://esid.org/Working-Parties/Registry/Diagnosis-criteria*.

## Review of the ESID/PAGID (1999) Criteria for CVID

According to the ESID/PAGID (1999) criteria, CVID is a diagnosis of exclusion (Table 1) (33). Patients were required to have an IgG level below 7–8 g/l [2 SD below the mean, or more accurately, below the 97.5th percentile, as immunoglobulin levels are not normally distributed (34)], as well as impaired vaccine challenge responses or absent isohemagglutinins and other secondary causes of hypogammaglobulinemia were to be excluded. The criteria did not include the characteristic histological features of the disorder or clinical sequelae resulting from the immune system failure (ISF).

Since that time, there have been major advances in the understanding of CVID. The majority of CVID patients have impaired memory B cell function with a reduction in switched memory B cells (9). In the last decade, there have been several genetic discoveries in patients with CVID-like conditions. Rare patients with monogenic defects of *CD19, CD20, CD21, CD81*, and *ICOS* have been identified (35–38). If identified by molecular diagnostic studies (39), these patients are, however, no longer classified as having CVID and are removed from further consideration of the disorder (40, 41). Genetic alterations from genome wide association studies, including copy number variations (42) and sequence variations in genes such as *TACI, BAFF* receptor, and *MSH5* may predispose to CVID. Mutations of *TACI, BAFF* receptor, and *MSH5* are also found in healthy individuals, but at lower frequency (28, 31, 43).

The ESID/PAGID (1999) criteria require IgG levels to be below 2 SD of the mean (Table 1). This means that 2.5% of the general population would meet this criterion (23). There is general agreement with the third ESID/PAGID (1999) criterion that other secondary causes of hypogammaglobulinemia including drug-induced disorders need to be excluded (22, 44, 45).

Perhaps the greatest difficulty with the ESID/PAGID (1999) criteria is the requirement for poor responses to vaccines. The ESID/PAGID (1999) criteria do not specify which vaccines should be used and there are significant variations in vaccine protocols in different studies (46, 47). Therefore, patients with trivial hypogammaglobulinemia with mildly impaired diphtheria antibody responses could be classified as having CVID. Poor responses to the diphtheria vaccine are common, even in normal persons, particularly with increasing age (23).

It is likely many patients with CVID have already generated vaccine-specific memory B cells following childhood immunization prior to LOAF. Therefore, assessing booster responses to childhood vaccines may be diagnostically misleading. This may explain why a significant minority of patients with presumed CVID have protective responses to tetanus toxoid and Pneumovax^®^ (48, 49). It is also debatable if the response to highly immunogenic proteins such as tetanus toxoid, administered with adjuvant, is a valid and reliable predictor of a protective response to pathogens *in vivo* (21). Specific concerns about using vaccines to assess the immune response are shown in Table 4.

**Table 4:** **Difficulties interpreting vaccine responses in CVID**.

Tetanus toxoid	Excellent immunogen (48)
	Presence of memory B cells from childhood tetanus vaccination can make responses difficult to interpret in CVID patients (21)
	Results should be compared to normal individuals (50)
	Uncertain validity of using simple antigens with adjuvant to gauge response to pathogens *in vivo* (28)
Diphtheria toxoid	Poor immunogen (23)
	Response should be compared with normal individuals (50)
	Questionable validity of using simple antigens to gauge response to pathogens *in vivo* (28)
*H. influenzae* type B (HIB)	There may be major differences between those who have not been immunized vs. those who have had this as part of their routine vaccines (51)
	Protective levels may not be logical: need to compare response with normal persons (52, 53)
Pneumovax^®^ (PPV)	Poor response in infants <2 years (54)
	Differences in responses between middle aged and elderly adults (55)
	Risk of unresponsiveness with repeated doses of PPV (56)
	Difficulties in measuring antibody responses
	Assays not standardized (8)
	Cross-reactive carbohydrates can interfere with the assay (57, 58)
	Some serotypes (serotype 3) are more immunogenic than others (6B and 23F) (54)
	No external quality assurance program for the assay
	Different platforms for pneumococcal antibody measurement may not be comparable (59)
	Disagreement about protective antibody levels (60–62)
	Mucosal protection may require higher antibody levels cf sepsis (63)
	Diagnostic criteria have not been defined: at least five different criteria in the literature (64, 65)
	Vaccine quality may vary: stability of conjugated vaccines. Lot to lot variation (54)
	Up to 18% of CVID patients respond to PPV (49)
	Use of Prevnar13^®^ as part of routine vaccines will make is difficult to measure responses to carbohydrates
Other vaccines	Not widely used, e.g., typhoid vaccine (54)
	Many CVID patients may respond, e.g., meningococcal vaccine (66)
	Experimental vaccines not approved by FDA ψ174 (67)
	Risk of adverse reactions: e.g. rabies vaccines (54)

*PPV, pneumococcal polysaccharide vaccine. PPV is administered to test response to T-cell independent carbohydrate antigens, while conjugated vaccines such as HIB and toxoids test responses to T-cell dependent antigens*.

The use of neoantigens such as rabies vaccine, typhoid vaccine, and experimental vaccines such as ψX174 to assess LOAF may be more predictive of an immune defect as patients are unlikely to have previously encountered these antigens (54). However, there have been concerns about risks associated with the rabies vaccine (54) and the typhoid vaccine is not yet widely used. The ψx174 vaccine has not been registered by the FDA and cannot be used in routine clinical practice (67).

The difficulty with diagnosis is illustrated in a recent study in which a new category of idiopathic primary hypogammaglobulinemia was proposed for symptomatic patients who did not meet the ESID/PAGID (1999) criteria for CVID (68). In spite of not meeting the ESID/PAGID (1999) criteria, many of these patients were treated with immunoglobulin. There is thus a discord between diagnosis and treatment in many patients with hypogammaglobulinemia/CVID.

The ESID/PAGID (1999) criteria do not specify the need for symptoms to establish the diagnosis. Therefore, important clinical manifestations and complications may not be obvious from different parts of the world, when using these criteria. This was illustrated in recent CVID studies, where there were wide variations in complications leading to different clinical phenotypes as well as bronchiectasis in countries within the European Union as well as the United States (4, 7, 69).

It is accepted that these criteria are relatively simple and could be used to diagnose patients in developing countries. The major difficulty is the interpretation of vaccine responses (Table 4), which is a pivotal component of these criteria. We are also concerned that the application of relatively simple criteria to a complex set of disorders will result in inaccuracies, as evidenced by the need for a new category of disorders, idiopathic primary hypogammaglobulinemia, described above. We are thus left with criteria that can be difficult to measure and interpret. Several eminent authors have expressed concern about the need for revised diagnostic criteria for CVID (70, 71).

## New Diagnostic Criteria for CVID

We recently proposed new diagnostic criteria for CVID (Table 2) (21). Treatment recommendations are closely linked to these diagnostic criteria (Figure 1). The Ameratunga et al. (2013) criteria emphasize both the clinical and laboratory sequelae of ISF in these patients (72).

In order to qualify as having probable CVID, patients must meet criteria in categories A, B, and C or D (Table 2). It is important for these criteria to be applied sequentially, as each subsequent category confers increasing specificity (Figure 1). A threshold of 5 g/l for IgG has been set for adults as this is the cut-off used in the French DEFI study (5). Patients must be older than 4 years of age and must not have a secondary cause for their hypogammaglobulinemia. If patients do not meet category A criteria, other criteria do not apply and a diagnosis of CVID cannot be entertained (Figure 1).

The most important feature of these criteria is clinical evidence of ISF (category B). Patients must have predisposition to infections and/or autoimmune disease, as a direct result of their immune defect. We have not been prescriptive about the numbers of upper or lower respiratory tract infections, as these will be influenced by the patient’s socio-demographic circumstances. While there may be debate about the specificity of each criterion in category B, the intention is to distinguish symptomatic patients from those who are well. These symptomatic patients must have laboratory evidence of immune dysfunction (category C) or the characteristic histological findings (category D) associated with CVID (Table 2).

None of the category C criteria are specific but in combination support the diagnosis of CVID. The majority of CVID patients will have a reduction of IgA and/or IgM (1, 4). Most will have impaired vaccine responses (49). It is, however, important to compare antibody responses to those of normal controls, as many CVID patients who have received their primary immunization series are likely to have developed memory B cells and are able to generate protective antibody levels. Using protective antibody levels as eligibility criteria for IVIG/SCIG is unacceptable in symptomatic patients as it will exclude a significant proportion of patients who will benefit from IVIG/SCIG treatment (48, 49).

Vaccine responses in normal controls have been published (50, 52, 53). Patients receiving a single booster dose of tetanus toxoid should achieve an antibody response of at least 1 IU/ml (50). Although a response of 1 IU/ml has been suggested following diphtheria immunization, our experience and original immunization studies show that this vaccine is much less immunogenic than the tetanus vaccine (50). Even otherwise healthy individuals may fail to reach this level, particularly persons over 50 years of age. The inclusion of diphtheria vaccine responses is thus problematic.

Adults and children receiving the *Haemophilus influenzae* type B (HIB) vaccine should reach antibody levels of at least 1.0 μg/ml rather than the protective level of 0.15 μg/ml (52, 53). Adults receiving the Pneumovax^®^ should achieve a protective level of 1.3 μg/ml for at least 70% of serotypes while children should reach 50% (64, 65). Adequate but transient vaccine responses are also included in category C criteria (21, 29). This may reflect *in vivo* failure of B cell memory in some patients.

We have included absent isohemagglutinins in these criteria providing the patient is not blood group AB (30). Although absent isohemagglutinins are part of the PAGID/ESID (1999) criteria, in our experience it is rare for a diagnosis of CVID to be made or for IVIG/SCIG to be prescribed, purely on the basis of absent isohemagglutinins in a patient with reduced IgG levels.

Most patients with CVID have impaired memory B cells and these constitute a diagnostic criterion in category C (9). It is, however, important for memory B cell subsets to be measured on at least two occasions, as we have shown the numbers can vary on repeat testing (73). Genetic predisposition (mutations of *TACI, BAFF* receptor, *MSH5*, etc.) to CVID is included in category C diagnostic criteria (42). This criterion is primarily intended for patients who may have been enrolled in clinical research studies. In the interim, we strongly discourage routine sequencing of these genes for diagnostic purposes until their utility is established.

Reduction or deficiency of IgG3 has been included in the new diagnostic criteria. There is considerable peer-reviewed literature suggesting IgG3 deficiency should be considered an important biomarker for a humoral immune defect (25, 26). We have included serological manifestations of significant autoimmunity in category C. This would include positive tests for lupus anticoagulant, Coombs test, etc. In most large series of CVID patients there are approximately 15% of patients who have severe autoimmunity and some have minimal infections in spite of their profound hypogammaglobulinemia. These patients will qualify as having CVID according to the Ameratunga et al. (2013) criteria, providing they have not been treated with rituximab.

Finally, some CVID patients have characteristic histological findings, which have been included in the Ameratunga et al. (2013) criteria (category D). These require biopsy for confirmation. In the context of primary (category A), symptomatic (category B) hypogammaglobulinemia, the presence of these relatively specific histological markers obviates the need to undertake vaccine responses, measure memory B cells, etc. Most of the histological criteria described in category D can occur with other disorders. Because category D will only apply if patients have already met category A and B criteria (Figure 1), this will confer specificity for CVID. Category C and D criteria may serve as an useful check list when assessing patients with hypogammaglobulinemia (21). We have shown that careful review of category C and D criteria may help to distinguish CVID from secondary causes of hypogammaglobulinemia (46, 74, 75).

As in the past, a diagnostic rectal biopsy could be undertaken to confirm the absence of plasma cells (19). Although a minority of CVID patients have plasma cells, the absence of these cells in a gut biopsy is a characteristic feature of CVID (21). Incorporating histological features in the diagnostic criteria may be useful where patients have already commenced IVIG/SCIG as there are risks in stopping treatment to assess vaccine responses.

We have designated a category of possible CVID for those patients meeting category A criteria (or AC or AD) but not category B criteria (Figure 1). Some asymptomatic patients with profound hypogammaglobulinemia may need to be treated with IVIG/SCIG as they may be at risk of severe viral infections or bacterial sepsis (28).

There are other patients who have mild hypogammaglobulinemia (IgG > 5 g/l) who are otherwise well or have only mild symptoms. We have termed these patients hypogammaglobulinemia of uncertain significance (HGUS) (21). In the future, it may be useful to sub classify HGUS patients depending on whether (sHGUS) or not (aHGUS) they have symptoms attributable to ISF. Prospective studies will indicate if the prognosis for these two sub groups is different.

The Ameratunga et al. (2013) criteria are intended primarily for clinical use and we therefore felt it was important to link them closely to treatment (Figure 1) (21). As with the Jones criteria for acute rheumatic fever, CVID no longer needs to be a diagnosis of exclusion.

## Revised ESID Registry (2014) Criteria for CVID

The revised ESID registry (2014) criteria for probable CVID have been recently released (http://esid.org/Working-Parties/Registry/Diagnosis-criteria) and are shown in Table 3. These are structured in a similar way to the Ameratunga et al. (2013) criteria but have not been given named categories. In contrast to the previous ESID/PAGID (1999) criteria, patients are required to have symptoms of their immune deficiency or a family history of antibody deficiency to be eligible for a diagnosis of CVID. Increased susceptibility to infection along with autoimmunity, unexplained polyclonal lymphoproliferation, or granulomatous disease qualifies patients for further consideration of CVID. This is similar to the Ameratunga et al. (2013) category B criteria, where a diagnosis of CVID cannot be made in the absence of symptoms (21).

Symptomatic patients are required to have a marked decrease of IgG as well as IgA and/or IgM. Again immunoglobulin levels 2 SD below mean is required for the relevant population. Immunoglobulin levels need to be repeated to confirm persistent reduction. This would exclude transient reductions in immunoglobulins that can sometimes be seen following viral infections or use of medications (44). Although a large number of patients with mild hypogammaglobulinemia will qualify for further investigation, the subsequent criteria will increase the specificity of the diagnosis.

Patients with hypogammaglobulinemia must then have either impaired antibody responses to vaccines and/or absent isohemagglutinins or reduced numbers of switched memory B cells for further consideration of CVID. Unlike the previous ESID/PAGID (1999) criteria, protective antibody levels are deemed to be required.

Like the Ameratunga et al. (2013) criteria, the revised ESID registry (2014) criteria do not specify which vaccines should be used. It is likely vaccine protocols will evolve with the availability and experience with new vaccines. As stated in Table 4, the increasing use of Prevnar13^®^ in routine childhood vaccine schedules will make assessing pneumococcal polysaccharide responses increasingly problematic. In contrast to the revised ESID registry (2014) criteria, the Ameratunga et al. (2013) criteria require vaccine responses to be compared to the normal population, as a significant proportion of presumed CVID patients have protective antibody responses to tetanus and Pneumovax^®^ (48, 49).

In contrast to the previous ESID/PAGID (1999) criteria, vaccine responses do not play such a pivotal role in the revised ESID registry (2014) criteria for CVID. Like the Ameratunga et al. (2013) criteria, it is therefore possible for patients to qualify as having CVID with normal vaccine challenge responses if they have either absent isohemagglutinins or reduced switched memory B cell numbers (Table 3). The requirement for reduced memory B cells in the revised ESID registry (2014) criteria is not mandatory and patients with absent B cells may still qualify for the diagnosis if they have impaired vaccine responses and/or absent isohemagglutinins.

As with all criteria described here, secondary causes of hypogammaglobulinemia must be excluded. The previous ESID/PAGID (1999) criteria specified 2 years as the eligible age for diagnosis, while the Ameratunga et al. (2013) and revised ESID registry criteria specify 4 years. The older age for diagnosis will help exclude monogenic defects as well as many cases of transient hypogammaglobulinemia of infancy. Unlike the ESID/PAGID (1999) and Ameratunga et al. (2013) criteria, the revised ESID registry (2014) criteria excludes severe T-cell defects from the spectrum of CVID, since these patients are deemed to have a combined immune deficiency. It is likely the genetic defect will differ from those with a largely humoral deficiency.

Granulomatous disease and lymphoproliferation are included in the initial set of clinical criteria in the revised ESID registry (2014) criteria (Table 3). This will require histological confirmation. Although not explicitly stated, our interpretation is that lymphoproliferation will include nodular lymphoid hyperplasia of the gut, nodular regenerative hyperplasia of the liver and lymphoid interstitial pneumonitis. These characteristic histological features and are included in category D of the Ameratunga et al. (2013) criteria. We assume an increase in CD21 low B cells may be included in the lymphoproliferation criterion of the revised ESID (2014) criteria. Like the Ameratunga et al. (2013) criteria, secondary causes for the histological lesions will be excluded by subsequent criteria. Nodular regenerative hyperplasia of the liver for example can occur with azathioprine treatment.

Like the Ameratunga et al. (2013) criteria, the revised ESID registry (2014) criteria may allow the diagnosis of CVID in patients who have already commenced IVIG/SCIG treatment. The presence of lymphoproliferation or reduced switched memory B cells will allow the diagnosis as long as other criteria are satisfied. This may obviate the need to stop IVIG/SCIG treatment to assess vaccine responses. Neither the Ameratunga et al. (2013) criteria nor the revised ESID (2014) registry criteria attempt to address the complex situation of hypogammaglobulinemia/CVID associated with malignancy. It can be very difficult to determine if hypogammaglobulinemia/CVID is the cause or the effect of malignancy.

Unlike the Ameratunga et al. (2013) criteria, the absence of plasma cells is not included in the revised ESID registry (2014) criteria. As indicated above, this is a useful feature in patients who have already commenced IVIG/SCIG replacement and may be identified by a diagnostic rectal biopsy. This is perhaps the most useful histological marker of CVID. The Ameratunga et al. (2013) criteria may be less useful if there are no characteristic histological lesions in patients who have already commenced IVIG/SCIG treatment. The emphasis on reduced switched memory B cells in the revised ESID registry (2014) criteria may allow the diagnosis in patients who have already commenced IVIG/SCIG. The revised ESID registry (2014) criteria require switched memory B cells to be below 70% of the normal population. Reference intervals will therefore have to be established for each laboratory. Given the variability in these cells, the Ameratunga et al. (2013) criteria require the assay to be repeated (73).

In comparison with the Ameratunga et al. (2013) criteria, transient vaccine responses are not included in the ESID registry (2014) criteria or breakthrough infections in spite of prophylactic antibiotics. Failure of vaccines to prevent infections, e.g., human papillomavirus is also not included in the revised criteria. The latter two may, however, be covered in the initial criterion of increased susceptibility to infection in the revised ESID registry (2013) criteria. Sequence variations in genes (*TACI, BAFF* receptor, *MSH5*, etc.) predisposing to CVID and IgG3 deficiency are not included in the revised ESID registry criteria (2014). Prospective studies will determine the value of these criteria (21).

The previous ESID/PAGID (1999) criteria had a category of possible CVID for patients not meeting the complete criteria for CVID (Table 1). The revised ESID registry (2014) criteria have a category of unclassified hypogammaglobulinemia for patients who do not meet all the criteria for CVID. Studies of cohorts of hypogammaglobulinemia patients will indicate if these categories are equivalent to patients with HGUS in the Ameratunga et al. (2013) criteria.

Like the previous ESID/PAGID (1999) criteria, diagnosis has not been linked to eligibility to IVIG/SCIG treatment in the revised ESID registry (2014) criteria, as these are intended for clinical research rather than therapy. However, it is inevitable these criteria will be used by clinicians to determine which patients will qualify for immunoglobulin treatment in the absence of specific national guidelines. Cohorts such as the NZ CVID/hypogammaglobulinemia study will be important in determining if the revised ESID registry (2014) criteria can be used to determine eligibility for treatment. It will be important to undertake prospective head to head comparisons of these criteria in hypogammaglobulinemic patients as we have done for memory B cells in CVID (73).

The latter two (Ameratunga et al. 2013 and the revised ESID registry 2014) criteria are based on the framework established by the ESID/PAGID 1999 diagnostic criteria. Most patients diagnosed with CVID will have substantially reduced IgG, reduction in other isotypes, impaired memory B cells, and impaired vaccine responses. The majority will thus qualify as having CVID by all three criteria. It should be noted that none of these criteria address partial antibody deficiency syndromes. This is an area that will need to be addressed in future studies.

It will be important to determine how these criteria perform in real-life clinical situations in ethnically diverse populations across the globe. We expect most if not all patients deemed to have idiopathic primary hypogammaglobulinemia, will qualify as having CVID, which will secure their eligibility for treatment with IVIG/SCIG (45, 68). Equally, it is hoped fewer asymptomatic patients with mild hypogammaglobulinemia will be treated with IVIG/SCIG.

Regardless of which criteria are used, it is essential that sound clinical judgment is exercised when diagnosing and treating these patients. It will be important to offer IVIG/SCIG for patients with bronchiectasis with higher levels of IgG for example (sHGUS) (76). Similarly, when assessing the cause of recurrent infections, there may be other contributing factors, which could be potentially treated in patients with hypogammaglobulinemia. The immune defect may not be the dominant predisposing factor for infections in some such individuals. Correcting functional or anatomical predisposing factors such as chronic sinus disease may reduce the number of infections and the patient may not need to be treated with IVIG/SCIG in spite of the hypogammaglobulinemia. This is why it is critical for these patients to be under the care of experienced clinical immunologists.

## Author Contributions

Rohan Ameratunga conceived the idea of the new diagnostic and treatment criteria for CVID. Maia Brewerton, Charlotte Slade, Anthony Jordan, David Gillis, Richard Steele, Wikke Koopmans, and See-Tarn Woon contributed references and made editorial changes necessary to support the manuscript.

## Conflict of Interest Statement

Rohan Ameratunga has received an unrestricted educational grant from Octapharma. The other co-authors declare that the research was conducted in the absence of any commercial or financial relationships that could be construed as a potential conflict of interest.
